# Diurnal gene expression patterns in retina and choroid distinguish myopia progression from myopia onset

**DOI:** 10.1371/journal.pone.0307091

**Published:** 2024-07-19

**Authors:** Richard A. Stone, John W. Tobias, Wenjie Wei, Xia Carlstedt, Lixin Zhang, P. Michael Iuvone, Debora L. Nickla

**Affiliations:** 1 Department of Ophthalmology, Perelman School of Medicine, University of Pennsylvania, Philadelphia, Pennsylvania, United States of America; 2 Penn Genomics and Sequencing Core, Perelman School of Medicine, University of Pennsylvania, Philadelphia, Pennsylvania, United States of America; 3 Department of Biomedical Sciences and Disease, New England College of Optometry, Boston, Massachusetts, United States of America; 4 Department of Ophthalmology & Department of Pharmacology and Chemical Biology, Emory University School of Medicine, Atlanta, Georgia, United States of America; Doheny Eye Institute/UCLA, UNITED STATES OF AMERICA

## Abstract

The world-wide prevalence of myopia (nearsightedness) is increasing, but its pathogenesis is incompletely understood. Among many putative mechanisms, laboratory and clinical findings have implicated circadian biology in the etiology of myopia. Consistent with a circadian hypothesis, we recently reported a marked variability in diurnal patterns of gene expression in two crucial tissues controlling post-natal refractive development ‐ the retina and choroid–at the onset of form-deprivation myopia in chick, a widely studied and validated model. To extend these observations, we assayed gene expression by RNA-Seq in retina and choroid during the progression of established unilateral form-deprivation myopia of chick. We assayed gene expression every 4 hours during a single day from myopic and contralateral control eyes. Retinal and choroidal gene expression in myopic vs. control eyes during myopia progression differed strikingly at discrete times during the day. Very few differentially expressed genes occurred at more than one time in either tissue during progressing myopia. Similarly, Gene Set Enrichment Analysis pathways varied markedly by time during the day. Some of the differentially expressed genes in progressing myopia coincided with candidate genes for human myopia, but only partially corresponded with genes previously identified at myopia onset. Considering other laboratory findings and human genetics and epidemiology, these results further link circadian biology to the pathogenesis of myopia; but they also point to important mechanistic differences between the onset of myopia and the progression of established myopia. Future laboratory and clinical investigations should systematically incorporate circadian mechanisms in studying the etiology of myopia and in seeking more effective treatments to normalize eye growth in children.

## Introduction

The coupling of the optics in the front of the eye with its axial length determines ocular refraction. During childhood, an active regulatory process synchronizes the expanding eye length with the corneal and lens powers; malfunctioning of this process yields refractive errors. In myopia (nearsightedness), the most common refractive error, the eye is relatively long for the optical power of the cornea and lens; distant images are blurred because their focus lies in front of the retinal photoreceptors [1]. While varying between countries, the prevalence of myopia is increasing markedly world-wide. It is reaching a prevalence in young adults of some 40–50% in the United States and Europe and 80–90% in regions of East and Southeast Asia [2–4]. By 2050, estimates suggest that some 50% of the world’s population will be myopic [5]. Besides blurred vision, myopia increases the risks for various retinal detachments, macula and retinal degenerations, glaucoma and certain forms of cataract; thus, myopia contributes to many blinding diseases of adulthood [6]. Notwithstanding many decades of clinical and basic research, many hypotheses and much speculation, the etiologic mechanism(s) for the altered eye development underlying myopia or for its increasing prevalence are unclear.

Convincing evidence, initially from animals but confirmed in children, has linked refractive development and myopia to visual input; and the controlling mechanism(s) localizes largely to the retina [7–10]. Not only in chicks but also in young mammals, for example, ipsilateral form-deprivation myopia follows the wearing of an image-degrading diffuser over an eye [11]. Visual input also influences the thickness of the choroid, the tissue adjacent to the retina. Interactions of the choroid with both retina and sclera are postulated to occur within a retina-to-choroid-to-sclera signaling cascade controlling overall ocular growth and refraction [12–14]. A vast number of signaling molecules, transcription factors, enzymes and biological pathways have been associated with myopia, but no coherent framework has yet emerged that clearly provides an explanation for the pathogenesis of clinical myopia [9].

Numerous laboratory and clinical findings have recently implicated circadian disruption in the development of myopia [9, 15–19]. Dimensions of both animal and human eyes fluctuate during the day, and these fluctuations seemingly affect overall eye growth and refractive development [13]. Experimental myopias of chick and mouse alter the retinal expression of clock and circadian rhythm-related genes [20–24]. In mice, Bmal1 clock gene knockout in retina produces myopia [25]. Melanopsin gene knockout in the retina of mice modifies normal eye development and increases experimental myopia [26]. Also in mice, obliterating intrinsically photosensitive retinal ganglion cells (ipRGCs) that project to the hypothalamus to regulate circadian rhythms curbs myopia [26, 27]. In young adult humans, short-term effects of light stimulation of ipRGCs suggest an interaction of ipRGC signaling with myopia stimulation [28]. Furthermore, the defocus and blurring of visual images that induce refractive errors in chick each modify the diurnal pattern of clock and circadian rhythm-related gene expression [29]. In human GWAS studies, the hundreds of specific genes and genetic loci associated with myopia and/or refractive error include genes mapping to genetic networks involving circadian control and light sensitivity [16, 19].

Because of these findings implicating circadian biology and because of diurnal changes in gene expression in many tissues [30, 31], we previously assayed gene expression in retina and choroid at six times over a day in chicks at the onset of unilateral form-deprivation myopia induced by unilateral diffuser wear [18], a well-established myopia model [32, 33]. We chose these two tissues because the visual signals regulating refractive development presumably originate in the retina and act at or through the choroid to modulate scleral growth and hence ocular size and refraction [11, 13, 14, 33, 34]. In both retina and choroid, we found surprisingly high variability of gene expression differences across the day comparing form-deprived to contralateral control eyes. Both in animals and in humans, the mechanisms acting at the onset of myopia likely differ from those during the progression of established myopia [35, 36]. Thus, we expanded our prior study [18]; but instead of assaying gene expression in retina and choroid during the first day of myopia initiation, we induced unilateral form-deprivation myopia in the same strain of chick and then assayed gene expression on the fifth day of continuous form-deprivation when myopia is both well-established and progressing rapidly [37, 38]. Not only did we find marked variability in gene expression in retina and in choroid over the course of a day during progressing myopia, but we also found that the altered diurnal gene expression during myopia progression differs markedly from that occurring at myopia onset.

## Materials and methods

### Animals and tissue harvesting

Except for a longer period of myopia duration, we followed the protocol we previously used to assess gene expression changes at myopia onset [18], studying unilateral form deprivation myopia in chicks (*Gallus gallus domesticus*) of the Cornell-K strain (a closed flock, random-bred for over 60 years). Newly hatched Chicks (n = 36 chicks) were reared for 12 days under a 12-hour light/12-hour dark cycle with illuminance of ∼300 lux in the cage (Phillips MAS LEDtube HF, 6500K; https://www.lighting.philips.com/main/prof/led-lamps-and-tubes/led-tubes/master-ledtube-instantfit-hf-t8/929001284202_EU/product). Then at ZT 0 (ZT, *zeitgeber* time; defined as lights on at ZT 0), a plastic image-degrading diffuser attached to a Velcro ring was fastened over the right eye using a complementary Velcro ring glued to the feathers around the eye. Diffusers were worn for four full 24-hour cycles, with the eye beneath the diffuser as “occluded” and the contralateral control eye with intact vision as “open.” Starting on day 5 of diffuser wear, chicks were killed by decapitation without anesthesia in timed groups, with chicks randomly assigned to time. Tissues were acquired at approximately ZT 0, 4, 8, 12, 16, or 20 hours (n = 6 chicks/time/condition), using a previously described tissue sampling procedure [29]. The duration of diffuser-wear corresponded to a time of established, rapidly advancing form-deprivation myopia in this strain of chick [37, 38]. For dissections during the dark phase, chicks were killed under a dim dark yellow light from a photographic safe light (Premier Model SL1012, Doran Manufacturing, Cincinnati OH, USA; ∼0.5 lux). After enucleation, eyes were put onto a bed of ice, hemisected at the ora serrata, and the vitreous was removed. Eyecups were put into Dulbecco’s Modified Eagle’s Medium under sterile and RNAse-free conditions, and the pecten was cut out using a razor blade. Using an oblique dissector/elevator (oblique ends 2mmx10mm, 6 ½”; item #160–720; George Tiemann & Co., Smithtown, NY), the entire extent of both the retina and choroid were dissected off of the sclera, and every attempt was made to remove any adhering RPE from both tissues. However, we cannot exclude the possibility that a small amount of RPE or RNA from RPE remained in some cases. To minimize RNA degradation, RNAase AWAY (ThermoFisher, Waltham, MA) was used to wipe the end of the periosteal elevator between the tissues. The tissues were immediately snap-frozen in liquid nitrogen, stored at −80°C until shipped on dry ice to the University of Pennsylvania. They were then kept at −80°C until processed further. The Institutional Animal Care and Use Committee (IACUC) of the New England College of Optometry approved the research (Approval #A4070-01). The research adhered to the ARVO Statement on the Use of Animals in Ophthalmic and Vision Research, was conducted in compliance with the relevant guidelines and regulations, and is reported in accordance with the ARRIVE (Animal Research: Reporting of In Vivo Experiments) guidelines.

### Library preparation and sequencing

The Next-Generation Sequencing Laboratory in the Penn Genomics and Sequencing Core (RRID:SCR_022383) extracted RNA from retina and choroid tissues with the QIAGEN microRNAeasy kit (QIAGEN, Germantown, MD, USA). RNA was stored at −80°C until shipped frozen to Azenta Life Sciences (South Plainfield, NJ, USA) for further processing and sequencing. Azenta had access only to the Penn-assigned integer labels of the samples but not to the experimental conditions of each sample.

Library preparation and sequencing were conducted by Azenta with PolyA selection and Illumina Sequencing. RNA samples were quantified using Qubit 2.0 Fluorometer (Life Technologies, Carlsbad, CA, USA). RNA integrity was checked using Agilent TapeStation 4200 (Agilent Technologies, Palo Alto, CA, USA). RIN (RNA Integrity Number) values were >9.0 for each sample, with mean RIN = 9.4±0.2 (mean±SD); see S1 Table for values of individual samples. Strand-specific RNA sequencing libraries were prepared by using NEBNext Ultra II Directional RNA Library Prep Kit for Illumina with 1 μg RNA/sample and following the manufacturer’s instructions (NEB, Ipswich, MA, USA). Briefly, the enriched RNAs were fragmented for 8 minutes at 94°C. First strand and second strand cDNA were subsequently synthesized. The second strand of cDNA was marked by incorporating dUTP during the synthesis. cDNA fragments were adenylated at 3’ ends, and indexed adapter was ligated to cDNA fragments. Limited cycle PCR was used for library enrichment. The incorporated dUTP in second strand cDNA quenched the amplification of second strand, which helped to preserve the strand specificity. The sequencing library was validated on the Agilent TapeStation (Agilent Technologies, Palo Alto, CA, USA), and quantified by using Qubit 2.0 Fluorometer (ThermoFisher Scientific, Waltham, MA, USA) as well as by quantitative PCR (KAPA Biosystems, Wilmington, MA, USA). The average insert size was approximately ∼560bp, as measured by Tapestation, with a mean molarity of 71.7nM/insert. The sequencing libraries were clustered on the flowcell. After clustering, the flowcell was loaded on the Illumina instrument (4000 or equivalent) according to manufacturer’s instructions. The samples were sequenced using a 2x150bp Paired End (PE) configuration. Image analysis and base calling were conducted by the Control software. Raw sequence data (.bcl files) generated the sequencer were converted into fastq files and de-multiplexed using Illumina’s bcl2fastq 2.20 software. One mismatch was allowed for index sequence identification. A total of 5.4 billion reads were mapped successfully to the chicken transcriptome, with a mean of 37.7 million mapped reads/sample and with a Mean Quality Score of (35.73±0.07)/sample (mean±SD; S1 Table).

### Data analysis

Since the nature of the data is similar, we followed the analysis technique we previously applied to our study of gene expression at myopia onset [18]. With Salmon [39], reads were mapped against the transcriptome described in Ensembl version 105.6 built on the chicken genome assembly GRCg6a. Applying several Bioconductor packages in R [40], transcriptome count data were annotated and summarized to the gene level with tximeta [41] and further annotated with biomaRt [42]. DESeq2 [43] was used for normalizations and statistical analyses.

DESeq2 was used to calculate normalized counts, variance stabilized counts and statistical analyses. For time-course analyses, a reduced model prioritized genes with unequal expression or differential expression across all time points. Included in the computations were baseMeans (the average normalized counts across all samples), p-values, and p-adj values [the p-values corrected for the false discovery rate (FDR) using the Benjamini-Hochberg method].

Based on the expression level of the HINTW (histidine triad nucleotide binding protein W; ENSGALG00000035998) gene, coded on the chicken female W chromosome, this study contained 15 female and 21 male chicks, with 2 or 3 female chicks and 3 or 4 male chicks at each individual time. Sex was removed as a source of variation when examining the factors of primary interest by including sex in the statistical model.

The main outcome analyses included: 1) Gene expressions in each condition (tissue, occluded eye, or open eye) were evaluated independently for changes over time, separate from changes in the other conditions. 2) Following an established analytical approach to unilateral experimental myopia, gene expression levels in occluded eyes were compared to those of its contralateral open control eyes at each time, as the log_2_-transformed ratio of the normalized means of [(occluded eyes) vs. (contralateral open eyes)] with p-adj values for statistical significance at each time. 3) To identify genes with varying expression over time in occluded eyes different from that in contralateral open eyes, we statistically modeled the interaction of treatment (occluded vs. open eye) with time for each tissue (i.e., occlVopen*time interaction). Differentially expressed genes occurring at more than one time during the day were found with Venn diagrams (http://www.interactivenn.net/index.html) [44].

**Fig 1:**
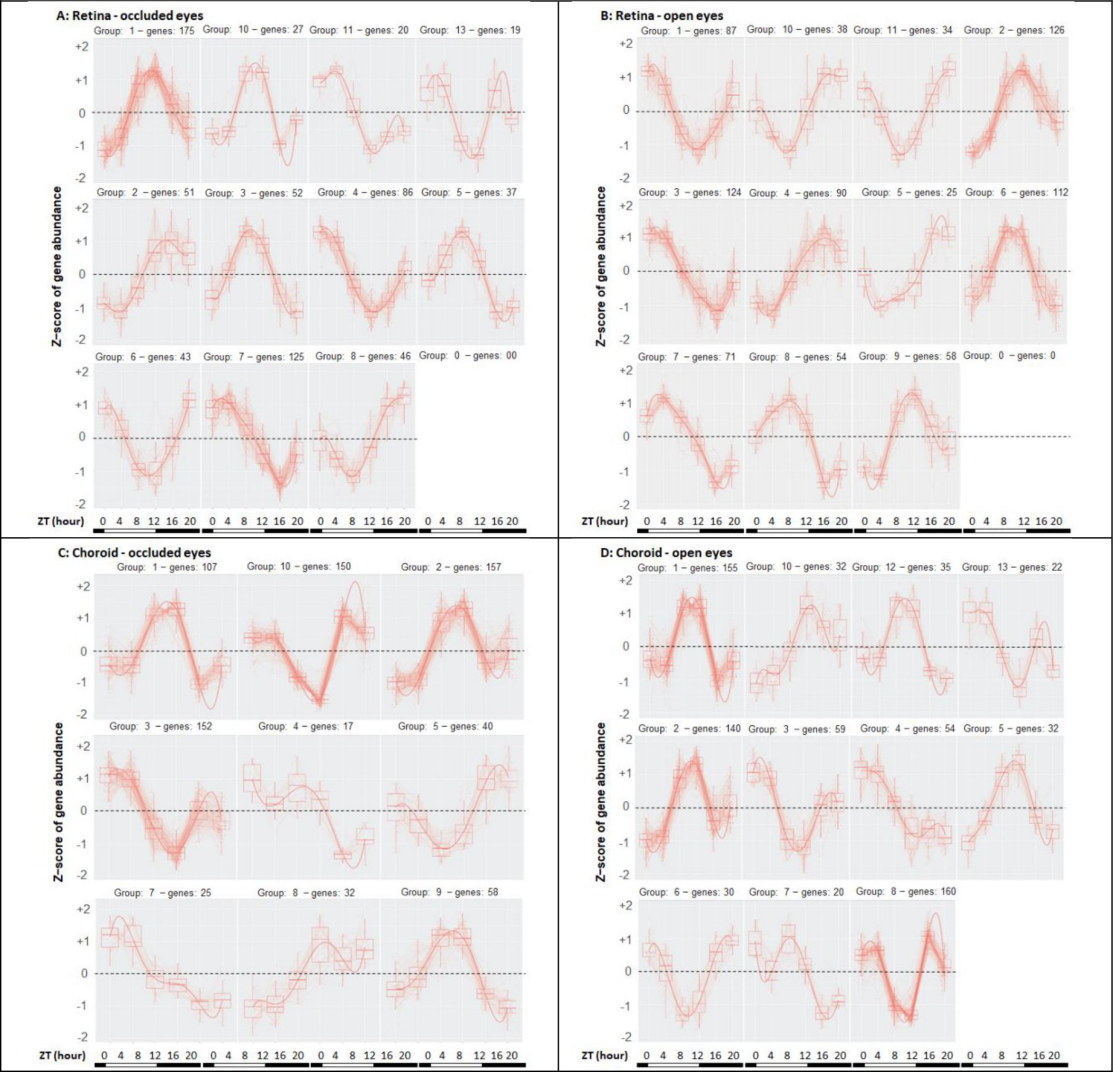
Variable gene expression patterns in retina and choroid during myopia progression. The clustered patterns of variable gene expression during the day are shown for the retina: occluded eyes (**A**) and contralateral open eyes (**B**); and for the choroid: occluded eyes (**C**) and contralateral open eyes (**D**). Subsets of the varying genes generated these expression patterns using statistical criteria described in the text, with the number of varying genes used to form each group above each panel. The densities of the tracings in each group reflect the number of genes conforming to each specific cluster. To illustrate the light:dark transitions, the bottom bars on each panel indicate the light phase (white bar) beginning at ZT0, and the dark phase (black bar) beginning at ZT12 and ending at ZT0. See S2A–S2D Table for the variability data of all genes over time for each tissue and eye, ranked by p-adj values. Abscissa: times of tissue sampling in ZT (hours). Ordinate or Z-scores: 0 = mean, with non-zero values corresponding to ± S.D.

**Fig 2:**
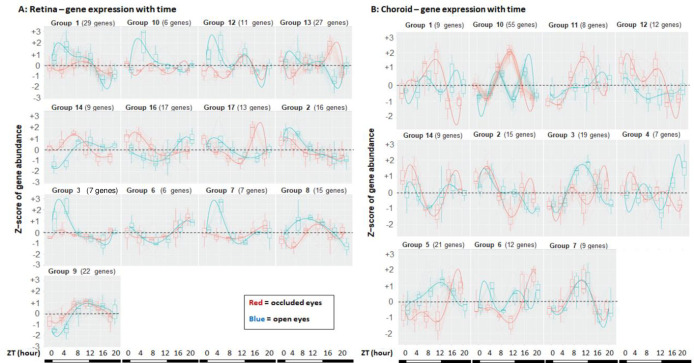
Gene expression patterns for the OcclVopen*time interactions during myopia progression, for retina and choroid. The groups into which genes clustered for the OcclVopen*time interactions over one day are shown for retina (**A**) and choroid (**B**). The limited number of genes meeting the criteria of p-adj<0.05 or p-adj<0.1 necessitated applying weaker statistical criteria to generate these groupings (see Results text). Most but not all interacting genes showed expression patterns demonstrated by these identified groups. The number of genes assignable to the individual groups appear above each panel. To illustrate light:dark transitions, the bottom bars on each panel signify the light phase (white bar) beginning at ZT0, and the dark phase (black bar) beginning at ZT12 and ending at ZT0. See S4A and S4B Table for the complete list of genes in each tissue in the occlVopen*time interaction category, including group assignment, gene names and descriptions, ranked by p-adj values. S1A Fig (retina) and S1B Fig (choroid) show heatmaps for the individual genes in the occlVopen*time interactions category meeting the p-adj<0.01 criterion. Abscissa: times of tissue sampling in ZT (hours). Ordinate or Z-scores: 0 = mean, with non-zero values corresponding ± S.D.

Using DESeq2 statistics [43], likelihood ratio tests (LRT) were calculated with a reduced model to prioritize genes exhibiting unequal expression across all time points. Using the degPatterns function from the DEGreport package [45], significantly changed genes across the day were clustered into groups demonstrating similar expression patterns; the clusters were displayed in graphs (Figs 1 and 2). The degPatterns function assigned directly the group names and orders in these graphs [45]. To identify pathway enrichments, Gene Set Enrichment Analysis (GSEA; v4.3.2) [46] evaluated pairwise comparisons between occluded and contralateral open eyes; GSEA uses the totality of genes measured to determine whether an *a priori* statistical ranking of those genes shows a positive or negative bias for a set of genes that corresponds to a pathway or functional group. The test is repeated for all gene sets in a collection. GSEA was performed in pre-ranked mode with the DESeq2 statistic as the ranking metric, and tested against the Canonical Pathways collection (C2:CP) of the Molecular Signatures Database (MSigDB; v2023.2.Hs; https://www.gsea-msigdb.org/gsea/msigdb). Based on preliminary comparisons of the gene sets in the Human Molecular Signatures Database, we selected the C2:CP curated collection because its pathway classifications supplied useful pathways (e.g., structure, inflammation, neurotransmission, peptide signaling, photoreception, etc.) to generate hypotheses for myopia mechanisms.

To compare chick genes identified in myopia progression to candidate human genes for myopia, we supplemented a recent summary of human candidate genes linked to myopia [20] with additional, non-overlapping genes from five other recent human genetic studies [47–51]. The chicken transcriptome (as its Ensembl gene identifications) was mapped via orthologs to the human gene symbols. A Chi-square test with Yates’ correction was used to compare the human gene list to the chick genes with statistically significant likelihood ratios in the occluded vs. open eye assessments and with the statistically significant likelihood ratios in the occlVopen*time interactions. Unless indicated otherwise, a value of p-adj or FDR less than 0.05 was judged statistically significant.

## Results

The present study on myopia progression parallels the design and analysis of our prior study of myopia onset [18]. For readers interested in comparing the two reports, selected findings at myopia onset are included here. As before [18], tissue was the main feature that differentiated gene expression in the samples. Sex (identified from HINTW gene expression; see Methods) provided a less robust differentiating feature, but sex was included in the statistical models to eliminate it as a contributing variable. The effects of eye and time were assessed in retina and choroid separately. Samples from retina generally displayed less variability than those from choroid.

### The variation of gene expression over time

For the ∼17,000 genes identified by Ensembl geneIDs, some 25% of genes in retina varied over the sampling times, and 31–37% varied in choroid, depending on whether the eye was occluded or open (Tables 1 and S2A–S2D). Since only one 24-hour day was studied and since we used neither constant light nor constant dark environments, we cannot distinguish between diurnal or circadian gene expression patterns and cannot separate diurnal/circadian effects from acute light effects [52].

**Table 1:** Number of genes with varying expression levels over time.

Eye	Retina		Choroid	
	Number of varying genes*	% **	Number of varying genes*	% **
Occluded eye	4223	24.6	6345	37.0
Open eye	4590	26.8	5267	30.7

Gene numbers with varying expression levels during the day for each tissue and eye, based on chicken Ensembl gene identifications. See S2A–S2D Table for complete lists of all identified genes with recordable p-adj values in each tissue and eye, sorted by p-adj values for their variation over time.

* Number of genes varying over the day in each tissue/eye with p-adj<0.05.

** Based on 17,136 total identified chicken genes.

To identify the various patterns of daily gene expression changes for each tissue and eye, we grouped the changes into distinct expression patterns over the day. Because of the large number of varying genes, we reduced this assessment to the most statistically significant subsets of the changing genes. For retina, the cutoff for the clustering models was p-adj = 1xe^-10^ (690 genes, occluded eyes; 865 genes, open eyes); for choroid, the cutoff was p-adj = 1xe^-7^ (759 genes, occluded eyes; 757 genes, open eyes). In retina, the variable genes clustered into 11 groups for both occluded and open eyes (Fig 1). In choroid, the variable genes clustered into 9 groups for occluded eyes and into 11 groups for open eyes (Fig 1). From these complex clusters of diurnal gene expression in each tissue and eye, the highest or lowest level of expression of individual genes occurred near the end of the light phase for many but not all gene expression patterns.

### Sampling time impacts occluded vs. open eye differences in gene expression

Tissue levels of gene expression, neurotransmitters or proteins are commonly compared between experimental and contralateral control eyes in seeking mechanisms underlying myopia, including form-deprivation myopia [9, 15, 18]. By the statistical criterion of p-adj<0.05, 121 genes in retina were differentially expressed at ZT04; and 341 genes in choroid were differentially expressed at ZT12. For either tissue at the other times, fewer or no genes were differentially expressed (Tables 2 and S3A and S3B). In the retina, the times with fewer gene expression differences occurred mostly in the dark phase or at the dark-to-light transition of ZT00; in the choroid, few gene expression differences occurred either in the light or the dark phases, with the preponderance of altered choroidal genes occurring at the end of the light phase, ZT12.

**Table 2:** Number of genes with occluded vs. open eye expression differences at each time, myopia progression.

Sampling time, ZT in hours	RETINA					CHOROID				
	Number of retinal genes	Direction of gene expression changes in occluded eye relative to open eye; number and % of genes				Number of choroidal genes	Direction of gene expression change in occluded eye relative to open eye; number and % of genes			
		Up		Down			Up		Down	
0	16	2	12.5%	14	87.5%	0	0	0.0%	0	0%
4	121	29	24.0%	92	76.0%	8	6	75.0%	2	25.0%
8	0	0	0.0%	0	0.0%	9	2	22.2%	7	77.8%
12	3	0	0.0%	3	100.0%	341	253	74.2%	88	25.8%
16	11	10	90.9%	1	9.1%	0	0	0.0%	0	0%
20	1	0	0.0%	1	100.0%	1	0	0.0%	1	100%
overall	14	1	7.1%	13	92.9%	2	2	100.0%	0	0.0%

Times and number of genes with expression differences between occluded and open eyes, p-adj <0.05.

“Up” = occluded/open eyes: + fold-change; “Down” = occluded/open eyes: − fold-change.

“overall” = genes with occluded vs. open eye differences that were similar in direction and magnitude at all time points (see text).

S3A (retina) and S3B (choroid) Table list by time the specific genes for each tissue with expression differences (p-adj<0.05), ranked by log_2_ fold change.

For retina or choroid, the genes in the “overall” category were identified by considering concurrently all replicates at all time points and prioritizing genes with occluded responses similar in direction and magnitude at all time points. The genes in the overall category were not required to differ significantly between occluded and open eyes at every time point. The overall category included only a small number of genes that differed statistically between occluded and open eyes over the full day in each tissue (Tables 2 and 3 and S3A and S3B).

**Table 3:** Overall category, retina, genes with similar differences over the day during myopia progression.

ETV5	MAFF	RAD54L2
RHOBTB2	DUSP4	LONRF1
MAFG	VPS26C	SPR
MIDN	EGLN3	CBARP
SPRY4		

Named genes in retina from the “overall” category of Table 2 with occluded vs. open eye differences that were similar in magnitude and direction at all time points (p-adj<0.05; see text). For choroid, only two genes, both identified by Ensembl gene IDs but non-named, met this criterion. See S3A and S3B Table for more information on specific genes, including the non-named genes in this category in both retina and choroid that are not shown here.

### Occluded vs. open eye differences interacted with time for a limited number of genes

We modeled the interaction of differential gene expression in occluded vs. open eyes with time (“occlVopen*time” category) for retina and for choroid. This analysis identified genes where the expression patterns in occluded eyes and contralateral open eyes differed statistically from each other across the day (Tables 4 and S4A and S4B). In seeking to identify the patterns of the differential gene expressions between the two eyes underlying the occlVopen*time interactions, the numbers of genes meeting either the p-adj<0.05 criterion (retina, n = 37; choroid, n = 7) or the p-adj<0.1 criterion (retina, n = 48; choroid, n = 12) were too few to cluster the gene expression patterns over time into groups. To increase the gene numbers for modeling, we selected a p-adj<0.4 for retina and approximately p-adj<0.797 for choroid that yielded 185 retinal genes and 176 choroidal genes that clustered into patterns where gene expression patterns differed between the two eyes. The genes in the occlVopen*time interaction category identified by these broadened statistical criteria are shown schematically in Fig 2 and are listed in S4A (retina) and S4B (choroid) Table. Depending on the modeled group, specific gene expressions in occluded eyes were higher or lower than the open eyes at some or most times. Occasionally, the relative gene expression levels became inverted between the two eyes over the day (e.g., Fig 2A, group 14). Heatmaps display the diurnal expression patterns of genes with statistically significant occlVopen*time interactions (p-adj<0.1) for retina and choroid (S1A and S1B Fig).

**Table 4:** Genes with occlVopen*time interactions during myopia progression.

RETINA			CHOROID
ETV4	MYBPC3	SMYD1	PRKCA
ETV5	TAMALIN	ACTC1	SLC4A2
ACTA1	MIDN	MYH7B	GPC4
MAFG	TNNI2	ANGPT2	FGFR4
MAFF	MYL1	PAK1IP1	CLEC19A
SPRY4	EPHA2	PER2	CHRM4
TNNT3	PISD	ARNTL	FXYD6
SPRED1	PCSK1	NOLC1	ARL13B
RHOBTB2	DUSP6	APOA1	HTRA1
MYL3	NPNT	LDB3	PHF19
RASL10A	TDP2	FAM89A	THBS4
OCM2	SPON1	MYH7	
SPRY2	VIP		
DUSP4	MYH1B		

Named genes with occluded vs. open eyes differences interacting with time over the day (occlVopen*time interaction) with statistical criteria of p-adj<0.10). See S4A and S4B Table for information not only for these genes but also for non-named genes and all 185 retinal genes and all 176 choroidal genes identified by the expanded statistical criteria described in the Results text, S4A and S4B Table include cluster (i.e., group) assignments, gene description, and statistical criteria for each tissue. S1A (retina) and S1B (choroid) Figs show heatmaps of the expression over time of those genes meeting the statistical criteria of p-adj<0.1.

### Only a few genes developed occluded vs. open eye expression differences at more than one time

**Fig 3:**
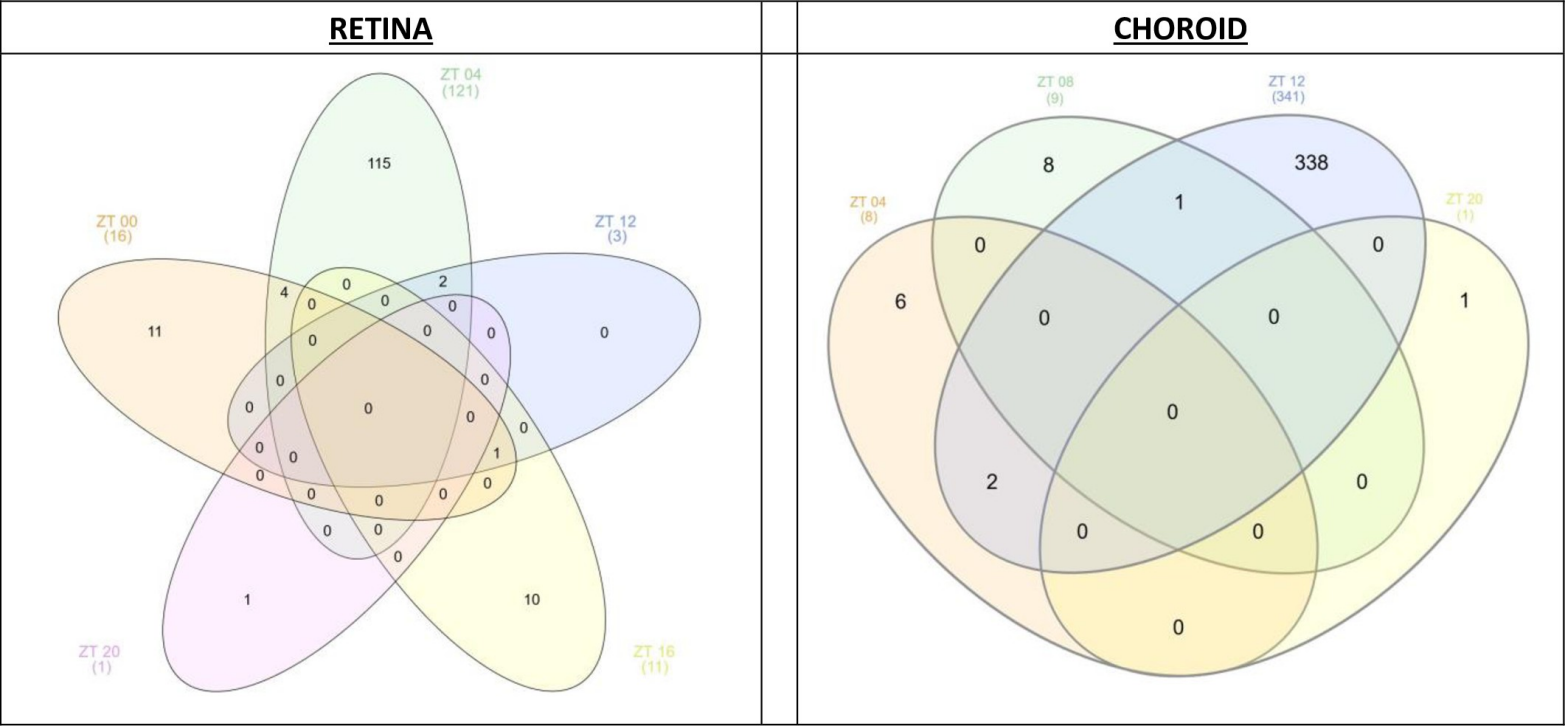
Venn diagrams: Genes with occluded vs. open eye differences at the sampling times during myopia progression, retina and choroid. The Venn diagrams identify the number of genes with differential expression levels at more than one time comparing occluded vs. open eyes in retina and in choroid, with the criterion of p-adj<0.05 for occluded vs. open eyes (see S3A and S3B Table). The different shapes of the Venn diagrams in the two panels occurred because the number of times with differentially expressed genes (Table 2) differed between retina (5 times) and choroid (4 times). S5 Table indicates times, the number of genes differentially expressed at the indicated times, the specific genes altered at more than one time, the directions of gene expression change and the log_2_ fold changes for both tissues.

Genes with differential expression levels between occluded vs. open eyes at more than one time were assessed with Venn diagrams (Fig 3) [44]. Regardless of how many genes showed occluded vs. open eye differences at a specific time (Table 2), very few genes exhibited occluded vs. open eye expression differences at more than one time: retina: 7 genes; choroid: 3 genes (Fig 3 and Tables 5 and S5). Most genes decreased at each common time point, although one gene in retina and two genes in choroid altered the direction of change depending on sampling time. Four retinal genes and one choroidal gene were altered at 2 consecutive times. Of the single gene in retina affected at 3 times, only two of the times were consecutive (S5 Table).

**Table 5:** Genes with occluded vs. open eye differences at more than one time during myopia progression.

Number of ZT times with between-eye differences in expression of individual genes	Number of genes, with names, showing differential gene expression at more than one time, by tissue	
	**Retina**	**Choroid**
**ZT times = 2**	6 genes	3 genes
**Individual gene names**	MAFF, PCSK1 DUSP4, EGLN3, VIP ENSGALG00000005011	AvBD1 ENSGALG00000051856 ENSGALG00000049118
**ZT times = 3**	1 gene	0 genes
**Individual gene name**	LMOD2	‐‐‐

By the criterion of p-adj<0.05 used for selecting individual genes and times for the Venn Diagrams, the table gives the number of genes with occluded vs. open eye differences at more than one time in each tissue. Only one gene was expressed at more than two times in occluded vs. open eyes in retina, but none in choroid. See S5 Table for gene descriptions, directions of gene expression changes and log_2_ fold changes at each ZT time for specific genes differentially expressed at more than one time.

S6A (retina) and S6B (choroid) Table list all gene data comparing occluded vs. open eyes at any p-adj level, with gene names and fold changes.

### Biochemical and signaling pathways during myopia progression

To identify signaling, structural and metabolic pathways potentially involved in progressing myopia in retina and choroid, we performed Gene Set Enrichment Analysis (GSEA) [46] on the relative expression of genes in occluded vs. contralateral open eyes (S7A and S7B Table). We found pathways in the GSEA report enriched in the occluded eyes relative to contralateral open eyes that corresponded to genes with increased expression in occluded relative to the contralateral eyes. We also identified pathways enriched in the open eyes relative to contralateral occluded eyes that corresponded to pathways from genes with decreased expression in occluded eyes relative to contralateral open eyes. GSEA uses unfiltered but ranked gene lists to detect pathways from genes moving in the same direction and identified pathways from each tissue and each time condition, even if few or no individual genes under some conditions met the statistical p-adj<0.05 criterion for differential gene expression. Based on an FDR<0.05 for pathway identification, we evaluated GSEA for the occlVopen*time interactions, for the overall category and for each time (see S3A, S3B, S4A, S4B, S7A and S7B Tables).

### Some differentially expressed chick genes corresponded to candidate genes for human myopia

We compared candidate human genes from recent publications (see Methods) [20, 47–51] to the differentially expressed genes identified in the progression of form-deprivation myopia in chicks (p-adj<0.05; S3A, S3B, S4A and S4B Tables). Only a limited number of candidate human genes corresponded to the differentially expressed genes in established, progressing chick form-deprivation myopia (Table 6). The retinal genes in chick corresponding to human genes were found mostly at chick light-phase sampling time ZT04, consistent with the preponderance of differentially expressed chick retinal genes at this time (Table 2). However, two overlapping chick/human genes also were identified in retina at sampling time ZT16, in the middle of the dark phase. The choroidal genes in chick corresponding to human genes were found only at sampling time ZT12, also consistent with the preponderance of differentially expressed chick genes in choroid at this time (Table 2). No genes in the occlVopen*time interaction or overall categories corresponded significantly with the human gene list.

**Table 6:** Common genes from occluded vs. open chick eyes and candidate human myopia genes.

Sampling condition for chick genes (ZT in hours)	Human candidate genes corresponding to differentially expressed genes in progressing form deprivation myopia of chick	
	**Retina** *	Choroid *
occlVopen*time	n.s.	n.s.
overall	n.s.	n.s.
0	n.s.	n.s.
4	NOG, SBSPON, GJD2, HIVEP3, PDE3A, PDE10A, PDE11A, CACNA1S, ENSGALG00000034119, ACTC1, ENSGALG00000054033	n.s.
8	n.s.	n.s.
12	n.s.	ECEL1, NEUROD6, OPN1LW, CLUL1, LRIT2, VSX1, GJD2, PAX6, RBFOX1, RD3L, RS1, ENSGALG00000050676, CABP4, GNB3, DSCAML1, GABRR1, BMP3, ENSGALG00000049411, FREM1, PER3, GJC2, DIS3L2, SARNP, GRIN2B, FGFR3, GDF11
16	INPP5D, EPHA2	n.s.
20	n.s.	n.s.

Chick genes with differences between occluded and open chick eyes for each sampling condition (p-adj<0.1 for occlVopen*time, S3A, S3B, S4A and S4B Tables; p-adj<0.05 for other conditions, S3A and S3B Table) that also were identified in human candidate human myopia genes obtained from several studies [20, 47–51].

occlVopen*time category = occluded vs. open eyes differences interacting with time.

overall category = occluded vs. open eye differences similar in magnitude and direction

at all time points.

* statistically significant overlapping genes, 2-tailed Chi-square test with Yates’ correction,

p<0.05.

n.s., no statistically significant overlap between chick and human myopia genes.

## Discussion

Consistent with the evidence linking refractive development with circadian biology described in the Introduction, the expression levels of many genes during established and progressing myopia depend on the time of day in both retina and choroid (Table 1 and S2A, S2B, S2C and S2D), with expression patterns varying by gene, tissue, and the eye’s occluded/open status (Fig 1). In terms of the myopia analysis of occluded vs. open eye comparisons, the central finding during progression is that gene expression differences between occluded myopic and contralateral open eyes also depend on tissue sampling time during the day (Table 2 and S3A and S3B). Most differentially expressed retinal genes were identified during the light phase (ZT00-ZT12), and the largest number of gene expression differences between occluded and open eyes during myopia progression occurred at ZT04. Most differentially expressed retinal genes during the light phase were downregulated in the occluded eye. In choroid, the overwhelming number of differentially expressed genes occurred at the end of the light phase (ZT12); most of these were upregulated in the occluded eye (Table 2).

Comparing myopia onset to myopia progression (S8 Table), most differentially expressed genes in retina occurred during the light phase (ZT00 through ZT12) and were downregulated in both conditions. In choroid, many more genes were differentially expressed throughout the day at myopia onset than during progression. Based on findings during myopia onset and/or progression, experimental studies of myopia pathogenesis thus need to include sampling time and myopia status when assaying for gene expression alterations because both can influence findings and bias the outcomes.

Only the current report and our prior report on myopia onset [18] incorporate time of day in sufficient detail to guide future research, particularly on circadian influences. Based on the statistical criteria used to identify differentially expressed genes for both tissues, S9A (retina) and S9B (choroid) Table describe all genes that 1) were differentially expressed at myopia onset; 2) were common to both onset and progression; and 3) were differentially expressed during myopia progression. Comparisons between onset and progression are discussed further below.

### OcclVopen*time interaction category

Modeling the interaction of treatment (i.e., occluded vs. open eye gene expression) with time provided the OcclVopen*time interaction categories (Fig 2 and Tables 4 and S4A and S4B). While more differentially expressed genes were identified from inter-eye comparisons at specific times in each tissue (Tables 2 and S3A and S3B), the OcclVopen*time interaction category incorporates data from many times and provides more robust comparisons of differentially regulated genes than the results of separately analyzed time points. We highlight here genes meeting the statistical cut-off of p-adj<0.1 (Table 4), even though broader statistical criteria were needed to generate enough genes to produce the groupings of Fig 2 (see Results text).

Retinal genes in the OcclVopen*time interaction category in myopia progression (Fig 2 and Tables 4 and S4A) include the circadian clock genes PER2 and ARNTL [29] and PCSK1, whose product processes prohormone and neuropeptide precursors [53]. The VIP gene conforms with the long-imputed role of VIP peptide in myopia; but the downregulation of VIP in progressing chick form deprivation myopia here and in chick myopia following the wearing of a minus spectacle lens (i.e., lens-induced myopia) [54] contrasts with its upregulation in primate and guinea pig myopia [55, 56], perhaps representing a species difference. Dermatan sulfate epimerase, a rate limiting enzyme in the synthesis of dermatan sulfate which has a role in tissue development and homeostasis [57, 58] also appears in this category. The protein product of NPNT, an extracellular matrix protein, regulates cell adhesion, differentiation and other functions [59]. ANGPT2 functions to influence vascular modeling and, at least in mammals, may impact development of the aqueous humor outflow pathway [60]; thus, NPNT may point toward a mechanism underlying the link between myopia and glaucoma [6]. Because the retina is a neural tissue, the expression of numerous genes linked to cardiac muscle (MYBPC3, ACTC1, MYL3, MYH7, MYH7B) or to skeletal muscle (MYL1, SMYD1, TNNI2, MYH1B, ACTA1, TNNT3) presents a conundrum for explaining a mechanism for myopia progression.

The choroid contained fewer genes in the OcclVopen*time interaction category during myopia progression than the retina (Fig 2 and Tables 4 and S4B). To highlight a few, the product of PRKCA is a protein kinase that influences proliferation, cell growth, inflammation, neurotransmitter release, circadian rhythmicity and phase setting [61]. While a role for fibroblast growth factor 4 (whose receptor is the product of FGFR4) is not known, other growth factors have been implicated in myopia, including fibroblast growth factor 2 [62]. Much work over centuries has implicated cholinergic signaling and cholinergic receptors in myopia [8, 9, 63], including the M4 muscarinic receptor subtype [64] which is the product of the ChRM4 gene in chick. The protein product of THBS4 influences cell-cell and cell-matrix interactions, tissue remodeling, synaptogenesis, angiogenesis and fibrosis among other activities [65]. FXYD6 encodes phosphohippolin, a modulator of Na,K-ATPase activity [66]; a potential control of eye growth and refraction by ion movements in the retina and choroid has previously been suggested [67, 68].

In the OcclVopen*time interaction category, the retina had several differentially expressed genes common to both myopia onset and progression (S9A and S10 Tables) [18], with both lists restricted to the p-adj<0.1 criterion. MAFF is a transcription factor. R-spondins enhance Wnt signaling [69], and Wnt signaling seemingly impacts myopia in humans and mice [70]. Most of the other common genes comparing OcclVopen*time category in retina have been discussed above. In choroid, only one gene (FXYD6, also discussed above) was shared in the interaction category of both conditions (S9B and S10 Tables). [18] In comparing the heatmaps during myopia progression (S1A and S1B Fig) with the heatmaps of the occlVopen*time interaction category at myopia onset [18], the common differentially expressed genes (S10 Table) between onset and progression tend to fluctuate less dramatically than some of the other the occlVopen*time genes. Whether these characteristics have mechanistic significance is uncertain.

### Overall category

The overall category lists the genes where the occluded vs. open eye comparisons were similar statistically in magnitude and direction throughout the day even though a statistically significant difference was not required at each sampling time. As a result of the statistical modeling, some genes were identified in each tissue for both overall and OcclVopen*time interaction categories. Despite the apparent statistical ambiguities in the overlap between these two categories, identification of these genes nonetheless has potential value in studying myopia pathogenesis. Only 14 genes in retina and 2 genes in choroid fell into the overall category during myopia progression. While only a small number of genes, their protein products may influence refractive development throughout the day.

Of the genes in the overall category, those in retina seemingly are most informative (Table 3 and S3A, S8 and S9A). To highlight a few besides transcription factors, SPR participates in the biosynthesis of tetrahydrobiopterin, a cofactor for tyrosine hydroxylase and tryptophan hydroxylase that have roles in dopamine and serotonin synthesis as well as functioning as an antioxidant [71]; dopamine and serotonin have each been implicated in myopia pathogenesis [72–74]. The protein products of MIDN and LONRF1 participate in ubiquination and protein degradation, among other potential actions [75, 76]. Two genes encode multifunctional proteins that interact negatively with mitogen-activated protein (MAP) kinase signaling: SPRY4, a protein inhibitor of MAP kinase signaling [77]; and DUSP4, a phosphatase acting at MAP kinase as well as at other proteins [78, 79]. MAP kinases are components of signaling pathways that govern general developmental phenomena such as cell proliferation and cell differentiation [80]. In the choroid, cartilage oligomeric matrix protein is the only gene product identified of the two differentially regulated genes in the overall category (S3B and S9B Tables); this large, complex extracellular protein exerts a variety of functions in the extracellular matrix [81].

The overall categories in the differentially expressed genes during myopia onset [18] and progression share only three genes in retina: LONRF1, RAD54L2 and DUSP4 (S9A Table). In choroid, no differentially regulated genes were common to both myopia onset and progression (S9B Table).

### Other differentially expressed genes at specific times

While less robust statistically than the two categories just discussed, additional genes identified at specific times merit some comment. Each tissue developed either up-regulated or down-regulated genes at many but not all times of day during myopia progression (Tables 2 and S3A and S3B). The overwhelming majority of differentially expressed genes in choroid occurred at ZT12 and most differentially expressed genes in retina occurred at earlier times, consistent with the notion of a time delay between at least some retinal signals and their actions at the choroid.

As examples for retina at ZT04 and ZT16 (Tables 2 and S3A), NOG encodes an inhibitor of bone morphogenetic proteins (BMPs); both NOG and BMPs have been implicated in myopia [22, 35]. GJD2, also implicated in human myopia [82], produces a gap junction protein connexin-36 that is regulated by dopamine [83, 84]. DIO2 has been previously associated with myopia [22], as have genes encoding urotensin (e.g., UTS2B) [22, 54]. The protein product of ANGP2 (angiopoitin-2) impacts vascular growth and inflammation [85], and angiopoitin-2 is a new target for retinal vascular disease [86]. NTNT encodes nephronectin, an extracellular matrix protein involved in cell adhesion and cell differentiation [59]. How, or even if, these protein products impact myopia requires further study.

In the choroid, the many genes differentially expressed at ZT12 (Tables 2 and S3B) during myopia progression impact such processes as inflammation, membrane signaling, ion channels and inflammation. While genes involving neural signaling and vision might have been expected to be found in retina, they here are found instead in choroid. Included in these genes are numerous genes related to receptors for the neurotransmitters GABA, glutamate and acetylcholine, several opsin genes and PRPH2 (also known as retinal degeneration slow, or RDS). Efforts during tissue dissection sought to minimize cross-contamination to the extent practical between the retina and choroid (see Methods). The genes for IGF-2 and several FGF receptor subtypes are identified. Also identified are DJD2 (seen in retina, above) and DJC2, both of which encode gap junction proteins [82]. The circadian clock gene PER3 and ASMT (whose protein product catalyzes the final step in melatonin synthesis) relate directly to potential circadian dysregulation in myopia, the hypothesis underlying this study.

Considering only the numbers of differentially regulated genes (S8 Table), there are surprisingly few common genes comparing myopia onset to myopia progression. Perhaps, there are relatively few signaling mechanisms common to both myopia onset and progression, and the common genes could be identifying common mechanisms essential to the characteristic ocular elongation in myopia (S9A and S9B Table). The limited overlap in the overall gene categories suggest that these genes and the processes they regulate (e.g., protein degradation or MAP kinase signaling in choroid) may be active throughout the day, and that only a few genes underlie time-independent signaling common to myopia onset and progression. Perhaps also, the distinct differentially expressed genes at myopia onset or during myopia progression reveal useful directions to favorably impact myopia, suggesting different interventions specifically for reducing the onset or the progression of myopia. Substantiating any of these notions, however, will require further research.

### Occluded vs. open eye differences at multiple times

Only a few individual genes developed statistically significant occluded vs. open eye differences at more than one time during myopia progression (Fig 3 and Tables 5 and S5). Individual genes may possibly have more influence on refraction if differentially expressed at multiple times in the day. Because of the 4-hour interval between tissue sampling times, however, gene expression differences at consecutive times may develop from a more pronounced effect at an intermediate time, rather than indicating a prolonged action. In retina, only 7 genes were differentially expressed at multiple times: of these, 3 were identified at non-consecutive times. In choroid, only 3 genes showed expression differences at multiple times: of these, 1 was identified at non-consecutive times (Fig 3 and Tables 5 and S5). The limited number of genes differentially expressed in each tissue at multiple times likely follows the concentration of differentially expressed genes to specific times (Table 2). Perhaps, the most interesting genes for the pathogenesis of myopia progression are PCSK1, DUSP and VIP in retina; each is discussed above. Many more genes were differentially expressed at multiple times in our previous study on myopia onset: 30 in retina and 89 in choroid [18]. Comparing the present progression study to myopia onset [18], only 5 genes were common to both studies in retina; and only one, in choroid (S11 Table). Like the broad perspective above on the limited common genes at myopia onset and during myopia progression, the limited common genes found at multiple times during myopia progression supports the hypothesis that myopia onset and progression may have different mechanisms.

### GSEA pathways

Of the many signaling, structural and metabolic pathways identified, those from the occlVopen*time and overall categories will be emphasized below (S7A and S7B Table). During myopia progression, pathways in retina in the occlVopen*time category enriched in occluded or open eyes include several pathways related to muscle, extracellular matrix, autophagy and the ARENRF2 anti-oxidant/detoxifying pathway [87]. The overall category in retina includes a number of pathways addressing oxidative metabolism, transport, protein processing, and nerve growth factor. Like individual genes, pathways in the choroid during myopia progression enriched in occluded or open eyes include many visual system and neural signaling pathways. In the occlVopen*time category, these include the cone, rhodopsin and phototransduction pathway, numerous neurotransmitter-related pathways (e.g., GABA, glutamate, norepinephrine, serotonin and synaptic vesicles), plus some involved in inflammation (e.g., interleukin 12) or cell biology. The overall category includes pathways enriched in occluded or open eyes for cones, phototransduction, some growth factors (IGF1, NGF), cell function, structure and inflammation.

Pathways identified previously at myopia onset are both similar and different from those found during myopia progression, using the same statistical criteria [18]. Retinal pathways found at myopia onset for the occlVopen*time category contrast with those during progression and include the rhodopsin pathway, Krebs cycle pathway and the mitochondrial complex 1 assembly pathway. In the overall category, retinal pathways at myopia onset include those involved in rhodopsin function, phototransduction, oxidative metabolism and various structural components. More similar to progressing myopia, the choroidal pathways at myopia onset for the occlVopen*time category contain neurotransmitter-related pathways (e.g., serotonin, dopamine, glutamate, norepinephrine, GABA, acetylcholine, etc.), cone pathways, metabolic pathways, as well as a few others. Among the choroidal pathways in the overall category at myopia onset are those related to neurotransmitters (e.g., GABA, serotonin, acetylcholine, GABA), cones, rhodopsin, visual transduction, ion channels, inflammation, etc. The pathway differences, especially for retina, further support the notion of substantial differences in the mechanisms of myopia onset and progression.

Relative to the circadian dysregulation hypothesis underlying this investigation, the circadian clock pathway is detected during myopia progression in retina at ZT04 and ZT20 but not in choroid; the clock pathway is not identified in either the occlVopen*time or overall categories of either tissue. The pathways from the occlVopen*time interaction and overall categories associated with photoreceptors and photoreceptor biology correspond to findings implicating photoreceptors in refractive error development in laboratory animals [67, 88] and in human genetics [16, 19, 89]. As in myopia onset [18], the many neurotransmitter-related pathways found in choroid during myopia progression suggest complex neuronal mechanisms in this tissue contributing not only to myopia onset but also to myopia progression. Besides our results in myopia onset and myopia progression, prior genome-wide assays of the retina have identified many complex pathways in chick [20–22, 90, 91] or mammalian [23, 24, 92] myopia. While a necessary consequence of the statistical nature of pathway identifications, the large number and variability of GSEA-generated pathways emphasize the complexity of the mechanisms underlying myopia onset and progression of established myopia.

#### Human vs. chick genes

In either retina or choroid, we found only a few differentially expressed genes represented during myopia progression among the many candidate genes for human myopia (Table 6). This limited overlap parallels our prior results at myopia onset [18] and the limited overlap in another study comparing chick retinal genes to those in human myopia at 4 hours and 24 hours of form deprivation [20]. The reason(s) for this limited overlap between chick and human myopia genes is uncertain. The anatomical parallels between form-deprivation myopia and human myopia, the induction of myopia in children from conditions that interfere with the visual axis (e.g., eyelid ptosis or corneal scarring) and similar anti-myopia responses to muscarinic antagonists in chicks and children [9, 93] suggest at least some similarity in underlying mechanisms. While requiring direct study, those human candidate genes overlapping with chick myopia genes may somehow be essential to myopia pathogenesis independent of species.

Among the retinal genes during chick myopia progression overlapping with human myopia genes in retina, EHPA2 encodes one of the Eph receptors, a large family of receptor tyrosine kinases that mediate many processes, such as cell-to-cell communication and cell attachment [94], as well as several phosphodiesterases and DJD2, a gap junction gene [82]. In the choroid, overlapping genes between chick and human include ECEL1, DJD2, PAX6, BMP3, FGFR3 and GDF11, all genes potentially interacting with signaling or growth mechanisms. The protein products of several genes have roles in neuronal biology signaling: CTNND2, GABRR1 and GRIN2B. Surprisingly, genes associated with retinal disorders (CLUL1, VSX1, RD3, RS1, and CABP4) and a cone pigment (OPN1LW) were identified in choroid but not retina of progressing chick myopia (Table 6). From single cell transcriptomics, several opsin genes also were recently found to be expressed in one or more choroidal cell types [95]. Refractive errors are associated with retinal diseases [89, 96], but the choroidal expression of these genes will require more study to understand. Refractive errors also occur in ocular diseases affecting other components of the eye, such as cornea or lens [1, 97], but whether our findings in retina or choroid relate to these conditions requires direct investigation.

As in our study of myopia onset [18], the overlap of human candidate myopia genes with chick myopia genes at discrete times in retina and in choroid during myopia progression suggests some type of connection to circadian biology that motivated this study. In both retina and choroid, some overlapping chick-human genes occur only during myopia onset, only during myopia progression or during both conditions. Perhaps, these distinctions between genes may help to identify mechanisms active at myopia onset, active at myopia progression or general pro-myopia mechanisms. As examples, the BMP’s and GABA signaling may be part of general myopia mechanisms, but the gap junction protein product of GJD2 may impact myopia progression. Such distinctions accessible in laboratory myopia models are hard to discern in clinical genetic risk factor studies. Such hypotheses would benefit from an improved understanding of the relationship between form-deprivation myopia and common human myopia.

### Circadian biology and clinical myopia

Many genes and pathways are linked by genetic studies to human myopia, including those involved with circadian rhythms [16]. Sleep disturbances, a potential indicator of circadian disruption, can occur in myopic children [98–100] but are not consistently detected [101–105]; the type of the sleep disorder also varies between studies [100, 106–108]. Sleep quality also may impact orthokeratology, a treatment for myopia [109]. The clock gene PER3 was identified as a rare variant in human myopia [49] (Table 6) and was found in the choroid during myopia progression at ZT12 (S3B Table); PER3 is involved in sleep regulation, non-visual responses to light and a variety of circadian responses [110], supporting potential roles for light and circadian biology in myopia. Increased time outdoors associates with a lower myopia prevalence but does not clearly impact myopia progression [111], suggesting perhaps that light and/or circadian effects may differently impact myopia onset and progression. Seeking a direct circadian effect on myopia, studies of children, early teenagers and young adults have so far resulted in conflicting findings of an association of myopia with systemic melatonin, an intrinsic mediator of circadian rhythms [112–114]. Reinforced by contemporary human genetics [16], environmental lighting has long been postulated to impact refractive development [15, 115–118]. The worsening problem of light pollution from ambient artificial light at night [119] can disrupt circadian rhythms and has been linked to sleep disorders in children [120] and perhaps myopia [121]. With recent concerns about putative pro-myopia effects of light from electronic screens [122], further investigation is needed to unravel the roles of light and sleep in myopia pathogenesis [123].

Besides the few common genes, many specific genes have previously been found and postulated as potential mediators of myopia both in experimental animals and in children [16, 19]. These many potential mediators of myopia onset and/or progression greatly complicate designing laboratory and clinical studies to study myopia mechanisms and to develop novel therapies. Perhaps because of the increasing biochemical and pharmacological complexity, novel myopia therapies increasingly assess specific wavelengths of light exposure, outdoor activities, optical devices and behavioral modification. Despite the clinical interest, only modest anti-myopia efficacy has resulted so far [124] with variable rebound of myopia on terminating these treatments [125].

Extending the results on the onset of form-deprivation myopia in chick, the current study finds a key role for time of day in potential retinal and choroidal signaling mechanisms during the progression of experimental myopia and further buttresses the notion that circadian biology exerts a central role in the mechanism(s) of myopia. Most available studies of myopia pathogenesis in children inadequately assess any circadian disorder or time of day, including the numerous light, optical or behavioral remedies proposed as potential myopia therapies. Identifying the type of any circadian disorder(s) causing/augmenting myopia and establishing any role for time of day in myopia pathogenesis would seem essential in clinical studies seeking the cause of myopia and improved anti-myopia interventions.

## Supporting information

S1 Fig A Retinal heatmap, myopia progression, occlVopen*time interaction. Heatmap of the log_2_ fold-changes in retina are shown across the sampling times for the occluded vs. open eye differences interacting with time (occlVopen*time interaction) during myopia progression. Key in upper right gives the magnitude of log_2_ fold-changes. The color indicates the direction of occluded vs. open eye gene expression differences–red, expression higher in occluded eye; blue, expression lower in the occluded eye. See Fig 2 and Tables 4 and S4A. lfc; log_2_ fold-change, preceded by the ZT of the tissue sampling time. Left ordinate: dendrogram relating the gene clusters. Right ordinate: gene name. B. Choroidal heatmap, myopia progression, occlVopen*time interaction. Heatmap of the log_2_ fold-changes in retina are shown across the sampling times for the occluded vs. open eye differences interacting with time (occlVopen*time interaction) during myopia progression. Key in upper right gives the magnitude of log_2_ fold-changes. The color indicates the direction of occluded vs. open eye gene expression differences–red, expression higher in occluded eye; blue, expression lower in the occluded eye. See Fig 2, Tables 4 and S4B. lfc; log_2_ fold-change, preceded by the ZT of the tissue sampling time. Left ordinate: dendrogram relating the gene clusters. Right ordinate: gene name.
(ZIP)

S1 Table RNA quantity and quality for each sample. The total amount of RNA extracted from each sample and two quality measures are shown for each chick, eye, tissue and ZT sampling time. *chick ID*, an arbitrary identification number used for this experimental series. *ZT*, Zeitgeber time of tissue sampling, in hours. *RIN*, RNA integrity number. *Mean Quality Score*, or Phred quality score (Q score) to assess accuracy of a sequencing platform.
(XLSX)

S2 Table Variability of gene expression across time during myopia progression. The variability of expression levels across time for all genes with measurable p-adj values during myopia progression, ordered by p-adj values, are shown for **S2A Table**) retinas of occluded eyes; **S2B Table**) retinas of contralateral open eyes**; S2C Table**) choroids of occluded eyes; and **S2D Table**) choroids of contralateral open eyes. *baseMean*, average normalized counts over all samples. *p-adj*, p-value corrected for the false discovery rate (FDR) with the Benjamini-Hochberg method.
(XLSX)

S3 Table **A. Comparisons of occluded to open eyes over time during myopia progression, retina**. The gene expression levels in occluded vs. contralateral open eyes over time in retina are provided for genes with between-eye differences meeting the criterion of p-adj<0.05, sorted by log_2_ fold-change. *ZT*, Zeitgeber time of tissue sampling, in hours. *overall*, retinal genes that differed statistically between occluded vs. contralateral open eyes over the entire day, identified by assessing all replicates at all time points simultaneously (see text, Table 2). *lfcSE*, standard error of the log_2_ fold-change. *p-adj*, p-value corrected for the false discovery rate (FDR) with the Benjamini-Hochberg method. **B. Comparisons of occluded to open eyes over time during myopia progression, choroid.** The gene expression levels in occluded vs. contralateral open eyes over time in choroid are provided for genes with between-eye differences meeting the criterion of p-adj<0.05, sorted by log_2_ fold-change. *ZT*, Zeitgeber time of tissue sampling, in hours. *overall*, retinal genes that differed statistically between occluded vs. contralateral open eyes over the entire day, identified by assessing all replicates at all time points simultaneously (see text, Table 2). *lfcSE*, standard error of the log_2_ fold-change. *p-adj*, p-value corrected for the false discovery rate (FDR) with the Benjamini-Hochberg method.
(ZIP)

S4 Table A. Group assignments of occlVopen*time interactions in retina during myopia progression. The inter-eye gene expression differences that interacted with time (i.e., occlVopen*time interaction), meeting the broadened statistical criteria described in the Results text, along with their cluster (group) assignments are shown for retina. The genes are ranked by p-adj values. *p-adj*, p-value corrected for the false discovery rate (FDR) using the Benjamini-Hochberg method. *cluster*, see Fig 2 for the representation of the patterns of the clusters (i.e., groups) and the text for the method of determining the occlVopen*time interactions. B. Group assignments of occlVopen*time interactions in choroid during myopia progression. The inter-eye gene expression differences that interacted with time (i.e., occlVopen*time interaction), meeting the broadened statistical criteria described in the Results text, along with their cluster (group) assignments are shown for choroid. The genes are ranked by p-adj values. *p-adj*, p-value corrected for the false discovery rate (FDR) using the Benjamini-Hochberg method. *cluster*, see Fig 2 for the representation of the patterns of the clusters (i.e., groups) and the text for the method of determining the occlVopen*time interactions.
(ZIP)

S5 Table Gene expression differences between occluded and open eyes at more than one time during myopia progression. The Venn Diagrams of Fig 3 provide the genes with between-eye expression differences with p-adj<0.05 at more than one time. The S5 Table lists the ZT times, the number and names of genes differentially expressed a more than one ZT time, the gene descriptions, the directions of gene expression changes and the log_2_ fold changes at statistically significant times. Retina, above; choroid, below. *ZT*, Zeitgeber time of tissue sampling, in hours.
(DOCX)

S6 Table **A. Complete data for occluded vs. open eye comparisons in retina during myopia progression**. The tables provide occluded vs. contralateral open eye gene expression differences over time in retina for all genes with the log_2_ fold-changes of the differences, sorted by p-adj values. *ZT*, Zeitgeber time of tissue sampling, in hours. *overall*, genes that differed statistically between occluded vs. contralateral open eyes over the entire day in retina, identified by assessing all replicates at all time points simultaneously (see text).*baseMean*, average normalized counts across all samples. *lfcSE*, standard error of the log_2_ fold-change. *p-adj*, p-value corrected for the false discovery rate (FDR) using the Benjamini-Hochberg method. **B. Complete data for occluded vs. open eye comparisons in choroid during myopia progression.** The tables provide occluded vs. contralateral open eye gene expression differences over time in choroid for all genes with the log_2_ fold-changes of the differences, sorted by p-adj values. *ZT*, Zeitgeber time of tissue sampling, in hours. *overall*, genes that differed statistically between occluded vs. contralateral open eyes over the entire day in choroid, identified by assessing all replicates at all time points simultaneously (see text). *baseMean*, average normalized counts across all samples. *lfcSE*, standard error of the log_2_ fold-change. *p-adj*, p-value corrected for the false discovery rate (FDR) using the Benjamini-Hochberg method.
(ZIP)

S7 Table **A. GSEA pathways in retina during myopia progression**. Pathway assignments were based on the relative expression of retinal genes in occluded eyes vs. contralateral open eyes. Two general types of GSEA pathways were identified: 1) pathways enriched in occluded eyes relative to the contralateral open genes; and 2) pathways enriched in open eyes relative to the contralateral occluded eyes, corresponding to decreased expression in occluded eyes. The genes generating the pathways included those identified for specific times of the day, the “overall” category (see S3A and S3B Table), and the occlVopen*time interaction (see Tables 4 and S4A and S4B). Pathways listed met the statistical criterion of FDR q-value<0.05. *GSEA*, Gene Set Enrichment Analysis. *ZT*, Zeitgeber time of tissue sampling, in hours. *NES*, normalized enrichment score. *FDR*, false discovery rate. **B. GSEA pathways in choroid during myopia progression.** Pathway assignments were based on the relative expression of choroidal genes in occluded eyes vs. contralateral open eyes. Two general types of GSEA pathways were identified: 1) pathways enriched in occluded eyes relative to the contralateral open genes; and 2) pathways enriched in open eyes relative to the contralateral occluded eyes, corresponding to decreased expression in occluded eyes. The genes generating the pathways included those identified for specific times of the day, the “overall” category (see S3A and S3B Table), and the occlVopen*time interaction (see Tables 4 and S4A and S4B). Pathways listed met the statistical criterion of FDR q-value<0.05. *GSEA*, Gene Set Enrichment Analysis. *ZT*, Zeitgeber time of tissue sampling, in hours. *NES*, normalized enrichment score. *FDR*, false discovery rate.
(ZIP)

S8 Table Myopia onset vs. myopia progression: Gene numbers with expression differences between occluded and open eyes. Myopia progression vs. myopia onset, summary of gene numbers with expression differences between occluded and open eyes at each time (p-adj<0.05). “UP” = occluded/open eyes: + fold-change; “DOWN” = occluded/open eyes: − fold-change. *ZT*, Zeitgeber time of tissue sampling, in hours. “overall” = genes with occluded vs. open eye differences that were similar in magnitude and direction at all time points (see text). S3A (retina) and S3B (choroid) Table of the current study and S2A (retina) and S2B (choroid) Table of the myopia onset study [18] include by time the gene names and gene descriptions for each tissue with expression differences (p-adj<0.05), ranked by log_2_ fold change.
(DOCX)

S9 Table **A. Distinct and common genes in retina, myopia onset vs. myopia progression**. Distinct and common genes in retina for the occlVopen*time interaction category, for the overall category and for each sampling time for occluded vs. open gene expression comparisons for myopia onset and myopia progression. Data derive from S2 and S3 Tables in our study of myopia onset [18] and from S3A, S3B, S4A and S4B Tables of current study of myopia progression. The statistical criteria for selecting genes were identical for the two studies: p-adj<0.1 for the occlVopen*time interaction categories, and p-adj<0.05 for the overall category and for the individual times. *ZT*, Zeitgeber time of tissue sampling, in hours. *overall*, genes that differed statistically between occluded vs. contralateral open eyes over the entire day. *baseMean*, average normalized counts across all samples. *lfcSE*, standard error of the log_2_ fold-change. *p-adj*, p-value corrected for the false discovery rate (FDR) using the Benjamini-Hochberg method. **B. Distinct and common genes in choroid, myopia onset vs. myopia progression**. Distinct and common genes in choroid for the occlVopen*time interaction category, for the overall category and for each sampling time for occluded vs. open gene expression comparisons for myopia onset and myopia progression. Data derive from S2 and S3 Tables in our study of myopia onset [18] and from S3A, S3B, S4A and S4B Tables of current study of myopia progression. The statistical criteria for selecting genes were identical for the two studies: p-adj<0.1 for the occlVopen*time interaction categories, and p-adj<0.05 for the overall category and for the individual times. *ZT*, Zeitgeber time of tissue sampling, in hours. *overall*, genes that differed statistically between occluded vs. contralateral open eyes over the entire day. *baseMean*, average normalized counts across all samples. *lfcSE*, standard error of the log_2_ fold-change. *p-adj*, p-value corrected for the false discovery rate (FDR) using the Benjamini-Hochberg method.
(ZIP)

S10 Table Myopia onset vs. myopia progression: Genes with OcclVopen*time interactions over the full day. Genes with occluded vs. open eye differences interacting with time over the full day (i.e., the occlVopen*interaction category) from Table 3 here and from Table 3 of our study of myopia onset [18]). Genes met the criterion of p-adj<0.1 in both studies (see texts). Named genes are listed, and Ensemble gene ID numbers are provided for non-named genes. See S9A (retina) and S9B (choroid) Table for more information including gene descriptions. Retina, top; choroid, bottom. Blue shading = genes that are common to myopia onset and myopia progression. * = the only non-named gene common to progression and onset, which appears in retina, is described as “SHC adaptor protein 4”.
(DOCX)

S11 Table Myopia onset vs. myopia progression: Genes with occluded vs. open eye differences at multiple times. Common genes with occluded vs. open eye differences at more than one time from the current report on myopia progression and from our study of myopia onset [18] for genes meeting the criterion of p-adj<0.05 in each study. For each gene, only times meeting this statistical criterion are shown. Retina, top; choroid, bottom. *ZT*, Zeitgeber time of tissue sampling, in hours.
(DOCX)
