# Supplementary figures and images for: Diurnal gene expression patterns in retina and choroid distinguish myopia progression from myopia onset
Source: PLoS One. 2024 Jul 19;19(7):e0307091. doi: 10.1371/journal.pone.0307091 (PMC11259283; doi:10.1371/journal.pone.0307091)

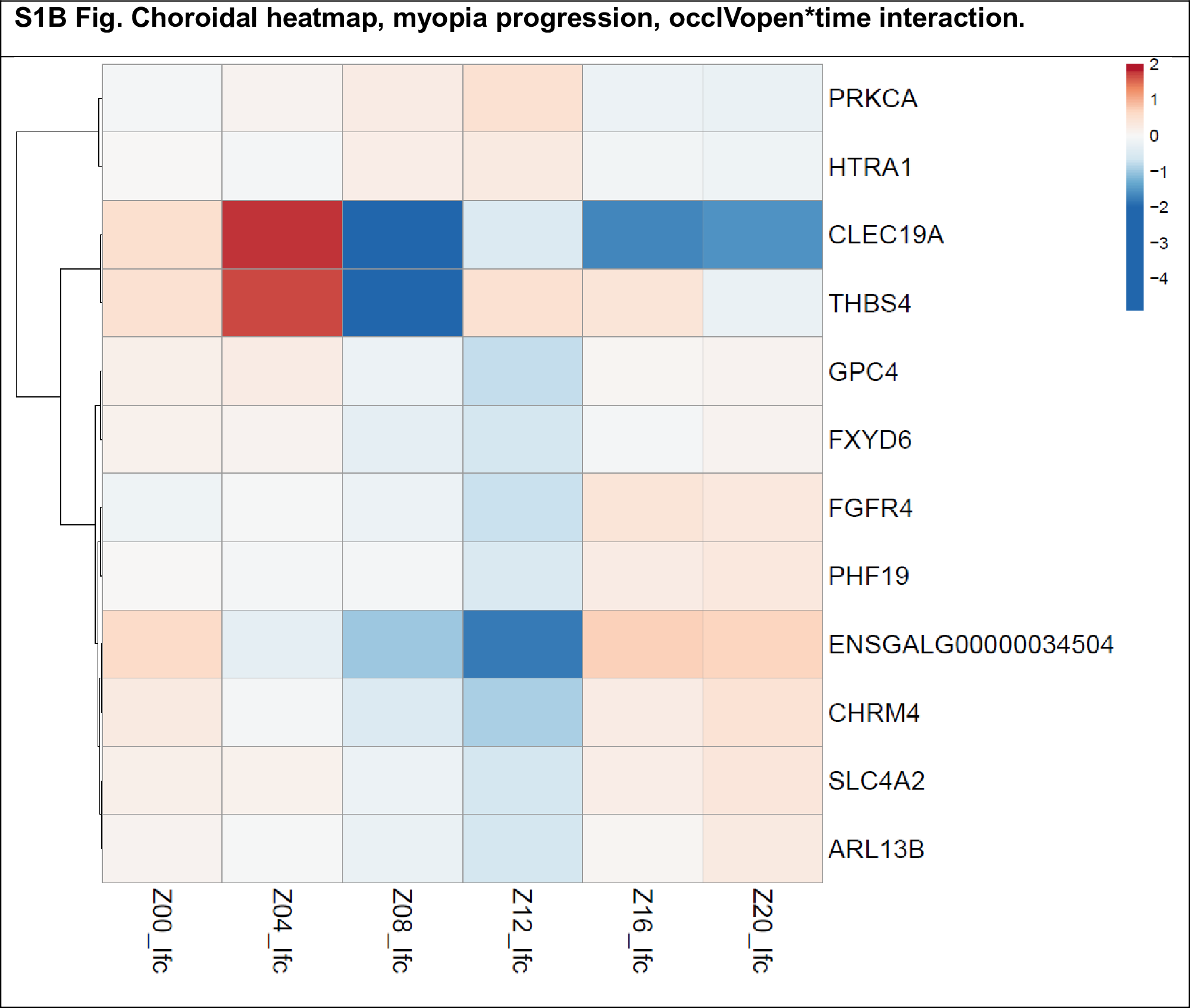

Supplement: S1 Fig — Heatmap of the log2 fold-changes in retina are shown across the sampling times for the occluded vs. open eye differences interacting with time (occlVopen*time interaction) during myopia progression. Key in upper right gives the magnitude of log2 fold-changes. The color indicates the direction of occluded vs. open eye gene expression differences–red, expression higher in occluded eye; blue, expression lower in the occluded eye. See Fig 2 and Tables 4 and S4A. lfc; log2 fold-change, preceded by the ZT of the tissue sampling time. Left ordinate: dendrogram relating the gene clusters. Right ordinate: gene name. B. Choroidal heatmap, myopia progression, occlVopen*time interaction. Heatmap of the log2 fold-changes in retina are shown across the sampling times for the occluded vs. open eye differences interacting with time (occlVopen*time interaction) during myopia progression. Key in upper right gives the magnitude of log2 fold-changes. The color indicates the direction of occluded vs. open eye gene expression differences–red, expression higher in occluded eye; blue, expression lower in the occluded eye. See Fig 2, Tables 4 and S4B. lfc; log2 fold-change, preceded by the ZT of the tissue sampling time. Left ordinate: dendrogram relating the gene clusters. Right ordinate: gene name. (ZIP) [file pone.0307091.s001.zip › S1B Fig. Choroidal heatmap, occlVopen-time.tif]

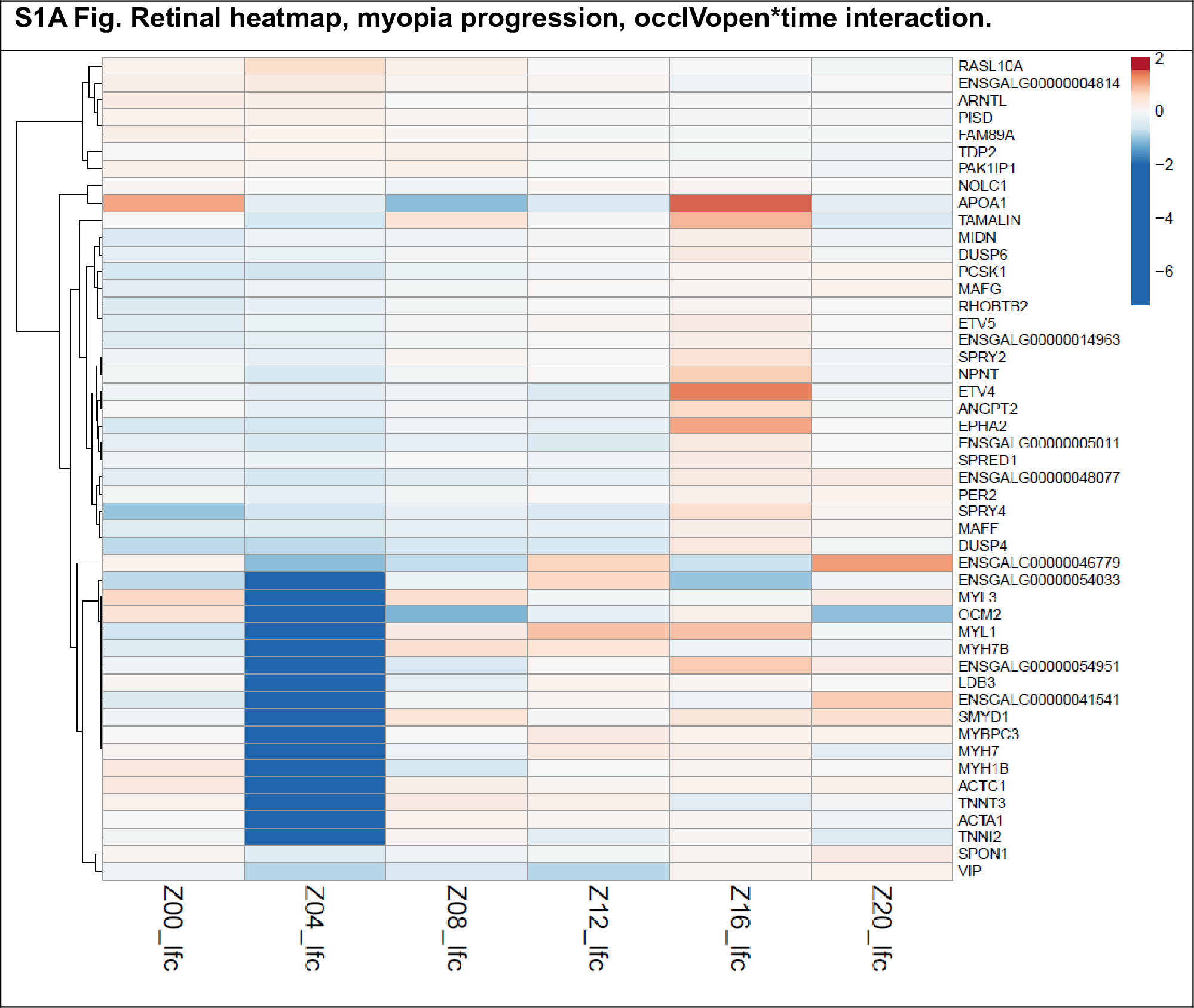

Supplement: S1 Fig — Heatmap of the log2 fold-changes in retina are shown across the sampling times for the occluded vs. open eye differences interacting with time (occlVopen*time interaction) during myopia progression. Key in upper right gives the magnitude of log2 fold-changes. The color indicates the direction of occluded vs. open eye gene expression differences–red, expression higher in occluded eye; blue, expression lower in the occluded eye. See Fig 2 and Tables 4 and S4A. lfc; log2 fold-change, preceded by the ZT of the tissue sampling time. Left ordinate: dendrogram relating the gene clusters. Right ordinate: gene name. B. Choroidal heatmap, myopia progression, occlVopen*time interaction. Heatmap of the log2 fold-changes in retina are shown across the sampling times for the occluded vs. open eye differences interacting with time (occlVopen*time interaction) during myopia progression. Key in upper right gives the magnitude of log2 fold-changes. The color indicates the direction of occluded vs. open eye gene expression differences–red, expression higher in occluded eye; blue, expression lower in the occluded eye. See Fig 2, Tables 4 and S4B. lfc; log2 fold-change, preceded by the ZT of the tissue sampling time. Left ordinate: dendrogram relating the gene clusters. Right ordinate: gene name. (ZIP) [file pone.0307091.s001.zip › S1A Fig. Retinal heatmap, occlVopen-time.tif]
