# Supplementary material for: Diurnal gene expression patterns in retina and choroid distinguish myopia progression from myopia onset
Source: PLoS One. 2024 Jul 19;19(7):e0307091. doi: 10.1371/journal.pone.0307091 (PMC11259283; doi:10.1371/journal.pone.0307091)
Supplement: S5 Table — The Venn Diagrams of Fig 3 provide the genes with between-eye expression differences with p-adj<0.05 at more than one time. The S5 Table lists the ZT times, the number and names of genes differentially expressed a more than one ZT time, the gene descriptions, the directions of gene expression changes and the log2 fold changes at statistically significant times. Retina, above; choroid, below. ZT, Zeitgeber time of tissue sampling, in hours. (DOCX) [file pone.0307091.s006.docx]

**S5 Table. Genes expression differences between occluded and open eyes at more than one time during myopia progression.**

| **Genes differentially expressed at more than one ZT time** | | **Gene**  **Name** | **Gene description** | **Direction of gene expression change at times (p-adj<0.05)** | | | | | | |
| --- | --- | --- | --- | --- | --- | --- | --- | --- | --- | --- |
| **ZT times of tissue sampling (hour)** | **Number of genes** |  |  |  |  |  |  |  |  |  |
|  |  |  |  |  | **ZT time and log_2_ fold change** | | | | | |
|  |  |  |  |  | 0 hr | 4 hr | 8 hr | 12 hr | 16 hr | 20 hr |
| **RETINA** |  |  |  |  |  |  |  |  |  |  |
| 0 & 4 | 4 | MAFF | MAF bZIP transcription factor F | all decrease | -0.44 | -0.44 |  |  |  |  |
|  |  | PCSK1 | proprotein convertase subtilisin/kexin type 1 | all decrease | -0.55 | -0.61 |  |  |  |  |
|  |  | DUSP4 | dual specificity phosphatase 4 | all decrease | -0.78 | -0.80 |  |  |  |  |
|  |  | EGLN3 | egl-9 family hypoxia inducible factor 3 | all decrease | -0.79 | -0.68 |  |  |  |  |
| 4 & 12 | 2 | ENSGALG00000005011 | SHC adaptor protein 4 | all decrease |  | -0.54 |  | -0.44 |  |  |
|  |  | VIP | vasoactive intestinal peptide | all decrease |  | -0.84 |  | -0.83 |  |  |
| 0 & 12 & 16 | 1 | LMOD2 | leiomodin 2 | increase ZT 0; decrease ZT 12 & 16 | +17.4 |  |  | -18.8 | -16.0 |  |
| **CHOROID** |  |  |  |  |  |  |  |  |  |  |
| 4 & 12 | 2 | AvBD1 | avian beta-defensin 1 | all decrease |  | -4.7 |  | -4.7 |  |  |
|  |  | ENSGALG00000051856 |  | decrease ZT4; increase ZT12 |  | -14.8 |  | +16.0 |  |  |
| 8 & 12 | 1 | ENSGALG00000049118 |  | increase ZT08;  decrease ZT12 |  |  | +16.4 | -16.7 |  |  |
