# Supplementary material for: Diurnal gene expression patterns in retina and choroid distinguish myopia progression from myopia onset
Source: PLoS One. 2024 Jul 19;19(7):e0307091. doi: 10.1371/journal.pone.0307091 (PMC11259283; doi:10.1371/journal.pone.0307091)
Supplement: S8 Table — Myopia progression vs. myopia onset, summary of gene numbers with expression differences between occluded and open eyes at each time (p-adj<0.05). “UP” = occluded/open eyes: + fold-change; “DOWN” = occluded/open eyes: − fold-change. ZT, Zeitgeber time of tissue sampling, in hours. “overall” = genes with occluded vs. open eye differences that were similar in magnitude and direction at all time points (see text). S3A (retina) and S3B (choroid) Table of the current study and S2A (retina) and S2B (choroid) Table of the myopia onset study [18] include by time the gene names and gene descriptions for each tissue with expression differences (p-adj<0.05), ranked by log2 fold change. (DOCX) [file pone.0307091.s009.docx]

| **S8 Table.** **Myopia onset vs. myopia progression, gene numbers with expression differences between occluded and open eyes.** | | | | | | | | | | |
| --- | --- | --- | --- | --- | --- | --- | --- | --- | --- | --- |
|  | **RETINA** | | | | | **CHOROID** | | | | |
| **Sampling time,**  **ZT in hours** | **Number of retinal genes** | **Direction of gene expression changes in occluded eye relative to open eye;**  **number and % of genes** | | | | **Number of choroidal genes** | **Direction of gene expression changes in occluded eye relative to open eye;**  **number and % of genes** | | | |
|  |  | **Up** | | **Down** | |  | **Up** | | **Down** | |
| **MYOPIA ONSET** | |  |  |  |  |  |  |  |  |  |
| overall | 24 | 3 | 12.5% | 21 | 87.5% | 32 | 20 | 62.5% | 12 | 37.5% |
| 0 | 28 | 4 | 14.3% | 24 | 85.7% | 39 | 24 | 61.5% | 15 | 38.5% |
| 4 | 62 | 10 | 16.1% | 52 | 83.9% | 27 | 9 | 33.3% | 18 | 66.7% |
| 8 | 119 | 53 | 44.5% | 66 | 55.5% | 673 | 205 | 30.5% | 468 | 69.5% |
| 12 | 21 | 5 | 23.8% | 16 | 76.2% | 51 | 23 | 45.1% | 28 | 54.9% |
| 16 | 4 | 0 | 0.0% | 4 | 100.0% | 96 | 64 | 66.7% | 32 | 33.3% |
| 20 | 0 | 0 | 0.0% | 0 | 0.0% | 193 | 156 | 80.8% | 37 | 19.2% |
| **MYOPIA PROGRESSION** | |  |  |  |  |  |  |  |  |  |
| overall | 14 | 1 | 7.1% | 13 | 92.9% | 2 | 2 | 100.0% | 0 | 0.0% |
| 0 | 16 | 2 | 12.5% | 14 | 87.5% | 0 | 0 | 0.0% | 0 | 0% |
| 4 | 121 | 29 | 24.0% | 92 | 76.0% | 8 | 6 | 75.0% | 2 | 25.0% |
| 8 | 0 | 0 | 0.0% | 0 | 0.0% | 9 | 2 | 22.2% | 7 | 77.8% |
| 12 | 3 | 0 | 0.0% | 3 | 100.0% | 341 | 253 | 74.2% | 88 | 25.8% |
| 16 | 11 | 10 | 90.9% | 1 | 9.1% | 0 | 0 | 0.0% | 0 | 0% |
| 20 | 1 | 0 | 0.0% | 1 | 100.0% | 1 | 0 | 0.0% | 1 | 100% |
