# Supplementary material for: Diurnal gene expression patterns in retina and choroid distinguish myopia progression from myopia onset
Source: PLoS One. 2024 Jul 19;19(7):e0307091. doi: 10.1371/journal.pone.0307091 (PMC11259283; doi:10.1371/journal.pone.0307091)
Supplement: S10 Table — Genes with occluded vs. open eye differences interacting with time over the full day (i.e., the occlVopen*interaction category) from Table 3 here and from Table 3 of our study of myopia onset [18]). Genes met the criterion of p-adj<0.1 in both studies (see texts). Named genes are listed, and Ensemble gene ID numbers are provided for non-named genes. See S9A (retina) and S9B (choroid) Table for more information including gene descriptions. Retina, top; choroid, bottom. Blue shading = genes that are common to myopia onset and myopia progression. * = the only non-named gene common to progression and onset, which appears in retina, is described as “SHC adaptor protein 4”. (DOCX) [file pone.0307091.s011.docx]

| **S10 Table. Myopia onset vs. myopia progression: Genes with occluded vs. open eye differences interacting with time (i.e., OcclVopen*time interactions) over the full day.** | | | | | |
| --- | --- | --- | --- | --- | --- |
| **RETINA** |  |  | **RETINA** |  |  |
| **MYOPIA ONSET** |  |  | **MYOPIA PROGRESSION** |  |  |
| VIP | DUSP4 |  | ETV4 | RHOBTB2 | TDP2 |
| DIO2 | TH |  | ETV5 | MYL3 | SPON1 |
| UNC5C | ACOX2 |  | ACTA1 | RASL10A | VIP |
| GLS2 | NOG |  | MAFG | OCM2 | MYH1B |
| PCSK1 | HBEGF |  | MAFF | SPRY2 | SMYD1 |
| NADK | RSPO2 |  | SPRY4 | DUSP4 | ACTC1 |
| SPON1 | NR2F2 |  | TNNT3 | MYBPC3 | MYH7B |
| PER2 | PWP1 |  | SPRED1 | TAMALIN | ANGPT2 |
| UTS2B | PRDM1 |  | ENSGALG00000005011 * | MIDN | PAK1IP1 |
| BMP2 | MGP |  | ENSGALG00000054951 | TNNI2 | PER2 |
| MAFF | COL9A2 |  | ENSGALG00000048077 | MYL1 | ARNTL |
| ENSGALG00000005011 * | CRHBP |  | ENSGALG00000054033 | EPHA2 | NOLC1 |
| ENSGALG00000011164 | PDE6B |  | ENSGALG00000014963 | PISD | APOA1 |
| ENSGALG00000007803 | G0S2 |  | ENSGALG00000041541 | PCSK1 | LDB3 |
| ENSGALG00000032749 | MFSD2A |  | ENSGALG00000046779 | DUSP6 | FAM89A |
|  |  |  | ENSGALG00000004814 | NPNT | MYH7 |

| **CHOROID** |  |  |  |  | **CHOROID** |
| --- | --- | --- | --- | --- | --- |
| **MYOPIA ONSET** |  |  |  |  | **MYOPIA PROGRESSION** |
| ATOH8 | VGLL3 | SNPH | TBC1D9 |  | PRKCA |
| ID3 | NSMF | SKIL | SYT9 |  | SLC4A2 |
| RGS16 | ADGRB2 | MSI1 | NOS1 |  | GPC4 |
| KRT40 | WASF1 | CDR2L | CNKSR2 |  | FGFR4 |
| ID2 | EML5 | RNF165 | GIN1 |  | CLEC19A |
| RGS8 | NMUR1 | CDC25A | SGO1 |  | CHRM4 |
| LBH | RAB3A | DIS3L2 | CD24 |  | FXYD6 |
| SLMAP | BTG2 | LECT2 | TMEM59L |  | ARL13B |
| TGFB3 | LIX1 | DEFB4A | IGF-I |  | HTRA1 |
| RASL11A | CYR61 | CATHL2 | ASTN1 |  | PHF19 |
| NOV | INTS11 | CATHL1 | CDH8 |  | THBS4 |
| ENSGALG00000046593 | ETV6 | LYG2 | CACNB3 |  | ENSGALG00000034504 |
| ENSGALG00000054904 | C1orf198 | AvBD1 | NCAPG2 |  |  |
| ENSGALG00000051492 | GPRIN2 | BD7 | NUSAP1 |  |  |
| ENSGALG00000006565 | NPTXR | ID4 | PISD |  |  |
| ENSGALG00000003203 | FXYD6 | AvBD6 | FOXM1 |  |  |
| ENSGALG00000027887 | SAMD11 | TMEM100 | ASTN1 |  |  |
| ENSGALG00000047192 |  |  |  |  |  |
