# Supplementary material for: Diurnal gene expression patterns in retina and choroid distinguish myopia progression from myopia onset
Source: PLoS One. 2024 Jul 19;19(7):e0307091. doi: 10.1371/journal.pone.0307091 (PMC11259283; doi:10.1371/journal.pone.0307091)
Supplement: S11 Table — Common genes with occluded vs. open eye differences at more than one time from the current report on myopia progression and from our study of myopia onset [18] for genes meeting the criterion of p-adj<0.05 in each study. For each gene, only times meeting this statistical criterion are shown. Retina, top; choroid, bottom. ZT, Zeitgeber time of tissue sampling, in hours. (DOCX) [file pone.0307091.s012.docx]

| **S11 Table. Myopia onset vs. myopia progression: genes with occluded vs. open eye differences at multiple times.** | | | | | |
| --- | --- | --- | --- | --- | --- |
| **Tissue and Gene Name** | **Gene Description** | **Myopia Onset** | | **Myopia Progression** | |
|  |  | **ZT times** | **Direction of change** | **ZT times** | **Direction of change** |
| ***Retina*** |  |  |  |  |  |
| MAFF | MAF bZIP transcription factor F | 8 & 12 | all decrease | 0 & 4 | all decrease |
| PCSK1 | proprotein convertase subtilisin/kexin type 1 | 0 & 8 & 12 | all decrease | 0 & 4 | all decrease |
| DUSP4 | dual specificity phosphatase 4 | 0 & 4 & 8 & 12 | all decrease | 0 & 4 | all decrease |
| ENSGALG0  0000005011 | SHC adaptor protein 4 | 0 & 8 & 12 | all decrease | 4 & 12 | all decrease |
| VIP | vasoactive intestinal peptide | 4 & 8 & 12 | all decrease | 4 & 12 | all decrease |
| ***Choroid*** |  |  |  |  |  |
| AvBD1 | Avian beta-defensin 1 | 4 & 8 | all decrease | 4 & 12 | all decrease |
